# Trim33 (Tif1γ) is not required for skeletal muscle development or regeneration but suppresses cholecystokinin expression

**DOI:** 10.1038/s41598-019-54651-8

**Published:** 2019-12-06

**Authors:** Cassie A. Parks, Katherine Pak, Iago Pinal-Fernandez, Wilson Huang, Assia Derfoul, Andrew L. Mammen

**Affiliations:** 1 National Institute of Arthritis and Musculoskeletal and Skin Diseases, National Institutes of Health, Bethesda, MD USA; 2 Johns Hopkins University School of Medicine, Baltimore, MD USA; 3 Faculty of Health Sciences, Universitat Oberta de Catalunya, Barcelona, Spain

**Keywords:** Transcriptomics, Muscle stem cells, Skeletal muscle

## Abstract

The expression of Trim33 (Tif1γ) increases in skeletal muscles during regeneration and decreases upon maturation. Although Trim33 is required for the normal development of other tissues, its role in skeletal muscle is unknown. The current study aimed to define the role of Trim33 in muscle development and regeneration. We generated mice with muscle-specific conditional knockout of Trim33 by combining floxed *Trim33* and Cre recombinase under the Pax7 promoter. Muscle regeneration was induced by injuring mouse muscles with cardiotoxin. We studied the consequences of Trim33 knockdown on viability, body weight, skeletal muscle histology, muscle regeneration, and gene expression. We also studied the effect of Trim33 silencing in satellite cells and the C2C12 mouse muscle cell line. Although Trim33 knockdown mice weighed less than control mice, their skeletal muscles were histologically unremarkable and regenerated normally following injury. Unexpectedly, RNAseq analysis revealed dramatically increased expression of cholecystokinin (CCK) in regenerating muscle from Trim33 knockout mice, satellite cells from Trim33 knockout mice, and C2C12 cells treated with Trim33 siRNA. Trim33 knockdown had no demonstrable effect on muscle differentiation or regeneration. However, Trim33 knockdown induced CCK expression in muscle, suggesting that suppression of CCK expression requires Trim33.

## Introduction

Trim33 (Tif1γ) belongs to the family of Tif1 proteins, which is characterized by a tripartite motif domain consisting of a RING domain, two B boxes, a coiled-coil domain, and a PHD finger and bromodomain^1^. The tripartite motif domain confers E3 ubiquitin ligase activity, which is dependent on the PHD-Bromo cassette’s chromatin reading activity. Trim33 has been shown to recognize histone modifications indicating poised chromatin, specifically an unmodified H4, H3K9me3, H3K18ac^2^. It has been implicated in regulating the TGFβ pathway, though this role is not completely understood. Trim33 is both a negative regulator of the canonical TGFβ pathway via ubiquitination of Smad4^1,3,4^, and to cooperate with the Smad4-Smad2/3 complex for transcriptional activation of master regulators^2^. Moreover, Trim33 is essential for hematopoiesis^3^ and the terminal differentiation of several tissues including the mammary glands^5^. Knocking out Trim33 results in embryonic lethality in mice^6^.

We have previously shown that Trim33 is upregulated in regenerating skeletal muscle fibers of mice following muscle injury^7^. In the current study, we sought to study its functional role in skeletal muscle *in vivo* via a conditional knockout mouse, *ex vivo* using satellite cells, and *in vitro* using siRNA in C2C12 cells.

## Methods

### Generation of muscle-specific Trim33 knockout mice

C57BL/6 TRIM33^*flox/flox*^ mice in which exons 2–4 are loxP-flanked had been generated previously^6^. C57BL/6 and Pax7-Cre^+/+^ (Jackson lab) were crossed to generate Pax7-Cre^+/−^TRIM33^*flox/flox*^ mice and Pax7-Cre^−/−^ TRIM33^*flox/flox*^ mice. This mating scheme allowed us to generate litters of Pax7-Cre^+/−^ TRIM33^*flox/flox*^ (TRIM33 KO) and Pax7-Cre^−/−^ TRIM33^*flox/flox*^ (WT) mice, which were used for muscle injury and regeneration experiments. For body mass measurements, a breeding scheme yielding heterozygote knockouts was used: Pax7-Cre^+/−^ TRIM33^*flox/+*^ and TRIM33^*flox/flox*^.

Progeny genotypes were assessed using primers flanking a TRIM33 LoxP site (Forward: CACCTGCCTCATTCTTACAGG Reverse: GGGAGGGAAAATCTGGCTGAA), and primers for universal Cre (Forward: TGATGAGGTTCGCAAGAACC Reverse: CCATGAGTGAACGAACCTGG). The null allele was amplified (Forward: GCACCTTGATGAGATCTTCCTCCTCC Reverse: GGGAGGGAAAATCTGGCTGAA) and sequenced using Eurofins DNA sequencing to ensure deletion of exons 2–4 (Supplementary Fig. 1). All mouse experiments were performed in accordance with protocol A014-07-03 authorized by the National Institute of Arthritis and Musculoskeletal and Skin Diseases/National Institutes of Health Animal Care and Use Committee.

### Body mass

Mice were weighed weekly from 14–80 days of age. Statistical comparisons between body masses of different genotypes were conducted using mixed modeling controlling by the age, gender, and the number of measurements of each mouse. Statistical analyses were done using STATA14.

### Cardiotoxin mouse muscle injury

The left tibialis anterior (TA) of mice at 10–12 weeks of age were injured by injecting 0.1 mL of 10 μM cardiotoxin (Calbiochem, catalog # 217504) resuspended in PBS. The left and right TAs of each mouse were harvested on days 3, 5, 10, 14, and 28 post-injury by euthanizing the mouse, dissecting the TA from TA tendon to knee, and freezing with pre-cooled methylbutane in dry-ice. Samples were stored at −80 °C. Because CTX production was discontinued during part of the time we were doing experiments, notexin (Latoxan, catalog # L8104) was used for muscle injury to induce satellite cell proliferation.

### Satellite cells

Skeletal muscles were dissected from both hind limbs and torn with forceps then digested with collagenase type 2 (Worthington, 2.5 U/ml) for 30 min at 37 °C. Following washing with PBS, a second digestion was performed with collagenase B (Roche Biochemicals 2.5U/ml) and dispase (Roche Biochemicals 2.4 U/ml) for 1 hour at 37 °C. Digestion reactions were stopped with 2 mM EDTA and cell preparation was diluted with PBS then passed through a 40 µm cell strainer. Cells were collected by centrifugation at 400 g for 5 min then counted. For fluorescence activated cell sorting (FACS), cell preparation was re-suspended in PBS supplemented with 15% heat-inactivated FBS at 1 × 10^7^ cells/ml. and incubated for 30 min at 4 °C with the following primary antibodies: anti-Cd11b, anti-CD31, anti-CD45 and anti-Sca -1 (BD Biosciences) conjugated to fluorescein isothiocyanate (FITC) in addition to anti-α7-integrin conjugated to phycoerythrin (PE)(MBL). Complete antibody information is described in Supplementary Table 1. To select for viability and exclude fiber debris, cells were co-stained with 1 mg/ml propidium iodide (PI) and 2.5 mg/ml Hoechst (Molecular Probes) and cells were resuspended at 1 × 10^7^ cells/ml immediately before sorting. For all antibodies, we performed fluorescence minus one control as well as single stain controls. Cell sorts were performed on an Influx or a FACSAria Fusion (Becton and Dickenson) equipped with three lasers using a 100 mm nozzle. Data was collected with FacsDIVA software and bioexponential analysis was performed using FlowJo 9.1 (Treestar) software.

### C2C12 TRIM33 siRNA

C_2_C_12_ cells are a murine-derived myoblast cell line obtained from ATCC. Proliferating cells were cultured in growth media (DMEM, 10% fetal calf serum, L-glutamine, and pen/strep.) When the cultures reached ~80% confluence, they were induced to differentiate by replacing growth media with differentiation media (DMEM, 2% horse serum, L-glutamine, and pen/strep).

For transfection experiments, 50,000 C_2_C_12_ cells were added to each well of a 6-well plate on day -3 and cultured in growth media overnight. On day -2, growth media was replaced by growth media without antibiotics. On day -1, 200 pmoles per well of Ambion Trim33 siRNA (#4390771) were transfected using Lipofectamine 2000 (Invitrogen) according to the manufacturer’s instructions. On day 0, the media was replaced with differentiation media without antibiotics.

### Mouse tissue histology and immunofluorescence microscopy

10 μM sections were used to make slides from frozen TAs on the Leica CM1860 cryostat at −20 °C, where TRIM33 KO and WT biological replicates from the same CTX time point were included on the same slide.

For fiber size quantification, sections were stained with a-laminin (Sigma L9393, 1:200), DAPI (Thermo Fisher Scientific 62247), and images were taken at 20x on the Zeiss Axiovert S100 scope. The slides were blinded, random images of each slide were obtained for each mouse, and all fibers were quantified. The Minimum Feret’s Diameter of each fiber was measured using ImageJ, was converted from pixels to μm using reticule measurements, and was used as a proxy for fiber size to control for angled sectioning. Comparisons between the number of fibers of a certain size between TRIM33 KO and WT were conducted using student’s t-tests, with a p-value of 0.05 used as a cutoff.

### RNA sequencing

35 × 10 μM sections were sliced from each TA on the Leica CM1860 cryostat at −20 °C and were immediately stored in a dry ice-cooled tube. Tissue was homogenized in 1 mL of TriZol using 1.4 mm ceramic beads on Betrin Technologies Precellys 24 Homogenizer for 3 × 15-second intervals, with 15 seconds between each homogenization. 0.2 mL of chloroform was used for extraction, and after centrifugation, the upper aqueous phase was combined with 0.5 mL of isopropanol. After centrifugation, the pellet was washed with 1 mL of 75% ethanol and resuspended in 40 μL RNase-free water. The final RNA was incubated at 55 °C for 10 minutes to remove any remaining ethanol. RNA integrity was tested by measuring the number equivalent (RINe) values using the Agilent Technologies 2200 Tapestation (mean RINe value = 7.9, SD = 0.9). Libraries were prepared using the NeoPrepTM system according to the TruSeqM Stranded mRNA Library Prep protocol (Illumina) and were sequenced on the Illumina HiSeq. 3000. Reads were demultiplexed using bcl2fastq 2.17.1, aligned using the STAR v.2.5^8^ and the abundance of each gene was quantified using StringTie v.1.3.3^9^. Differential expression using raw counts was performed using DESeq.^10^. The Benjamini-Hochberg correction was used to adjust for multiple comparisons and a corrected p-value (q-value) of 0.05 or less was considered statistically significant. For visualization purposes, gene-expression values (Transcripts Per Kilobase Million [TPM]) values were log-transformed (logTPM: log2[TPM + 1]) and data were processed using Python with the packages Numpy, Pandas, and Seaborn. All the RNAseq experiments were performed in male mice.

### RT-qPCR

RNAs from post-injury Trim33 KO and WT were collected for RNA Seq. cDNA was synthesized from 100 ng of total RNAs by using High capacity cDNA reverse transcription kit (Applied Biosystem, Thermo Fisher Scientific, 4368814). Quantitative RT-PCR was performed by using predesigned mouse CCK primer pairs (Invitrogen, USA, forward 5′- AAGAGCGGCGTATGTCTGTG -3′ and reverse 5′-CATCCAGCCCATGTAGTCCC-3′) and the housekeeping primer was mouse 18 S (forward 5′-GGACCAGAGCGAAAGCATTTG -3′ and reverse 5′-GCCAGTCGGCATCGTTTATG-3′). 2X Power SYBR Green PCR Master mix (Applied Biosystem, Thermo Fisher Scientific, 4367659) was used to amplification according to the manufacturer’s protocol in an Applied Biosystems ViiA7 by Life Technologies.

## Results

### Trim33 KO mice have lower body mass but no impairment of muscle regeneration

**Figure 1:**
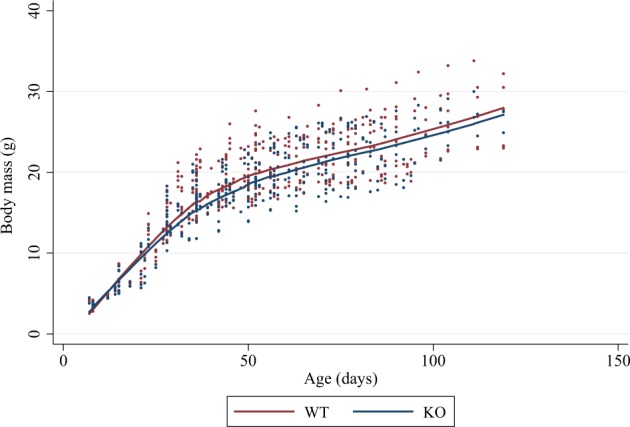
Decreased body mass in Trim33 knockout mice. Lowess curves and individual point measures of body masses (grams) of Trim33 WT (brown) and KO (blue) showing an ~7% reduction in the body mass of KO mice.

We measured the body mass of Trim33 KO and WT mice from 14–80 days of age (n = 71, 689 measurements). Using multilevel mixed regression models, we observed that independent of the age or gender of the mice, Trim33 KO mice weigh 1.3 g (~7%) less than WT mice (p = 0.004) (Fig. 1).

**Figure 2:**
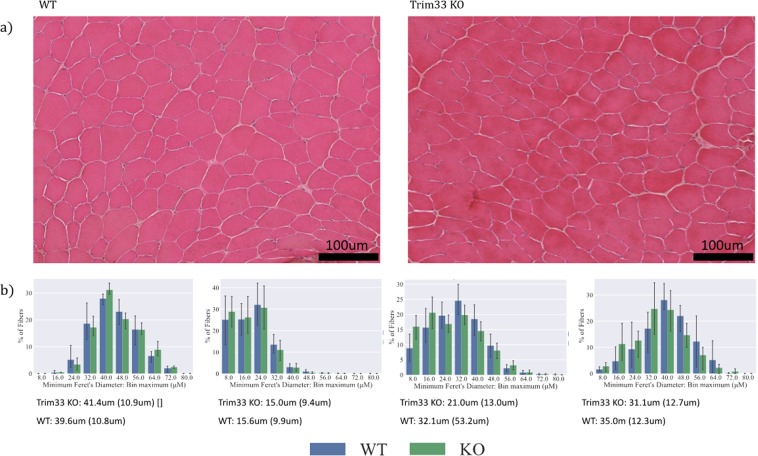
Normal skeletal muscle histology during regeneration in Trim33 knockout mice. (**a**) H&E appearance of WT and Trim33 KO muscle biopsies before cardiotoxin (CTX) injection. (**b**) Fiber size histograms in Trim33 KO (green) and WT (blue) mouse muscle before injury and at days 5, 14, and 28 after CTX injury showing no significant differences in muscle fiber sizes. Below each histogram is shown the mean and the standard deviation of the fiber size for each group. Fiber size histograms in Trim33 KO and WT mouse muscle before injury included 3 mice per group (mean = 395 fibers/mouse), at day 5 included 7 mice per group (mean = 810 fibers/mouse), at day 14 included 8 mice per group (mean = 750 fibers/mouse), and at day 28 included 3 mice per group (mean = 308 fibers/mouse).

To determine whether the decreased body mass was a result of decreased muscle mass, we examined the morphology of the Trim33 KO skeletal muscles. TA muscle sections stained with H&E from Trim33 KO mice had similar morphology to those of age-matched WT mice. Furthermore, quantification using the minimum Feret’s diameter (3 specimens per group, mean = 395 fibers/specimen) demonstrated that there were no significant differences in the myofiber size of KO mice (mean 41.4 um, SD 10.9 um) compared to WT mice (mean 39.6 um, SD 10.8 um) (Fig. 2). Also, we measured the weight of the forelimb (13KO, 12WT) and TA (8KO, 8WT) muscles and did not find a significant difference between groups (p = 0.114) adjusting by age and type of muscle.

To determine whether Trim33 KO impairs skeletal muscle regeneration, we used a CTX injury model. The Trim33 KO mouse muscle had a similar histological appearance as the WT skeletal muscle at all times following the injury and during the regeneration process. Consistent with this, there was no difference in myofiber size between KO and WT mice during muscle regeneration (Fig. 2).

Expression levels of genes involved in skeletal muscle differentiation, as well as adult muscle structural proteins, were similar in WT and KO muscle biopsies before and after muscle injury. (Supplementary Fig. 2). These gene sets were also not differentially regulated in satellite cells obtained from KO versus WT mice. Furthermore, these genes were not differentially expressed in C2C12 cells treated with Trim33 siRNA compared to those treated with control siRNA (all q > 0.05). Likewise, key TGFβ pathway genes were not differentially expressed in WT compared to KO skeletal muscle (Supplementary Fig. 3), WT versus KO satellite cells, or C2C12 cells with or without Trimm33 siRNA treatment (all q > 0.05).

### TRIM33 knockdown induces cholecystokinin overexpression

**Figure 3:**
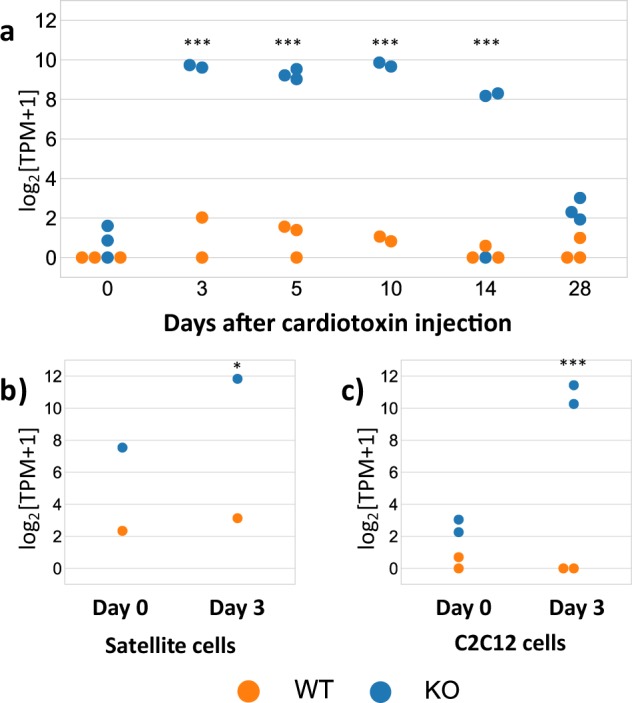
Overexpression of cholecystokinin with Trim33 depletion. Evolution of cholecystokinin (CCK) expression levels (log_2_[TPM + 1]) (**a**) before and after cardiotoxin injury in the skeletal muscle of WT vs KO mice, (**b**) in satellite cells harvested from WT vs. Trim33 KO mice before (day 0, myoblast) and after (day 3, myotubes) differentiation, and (**c**) in C2C12 cells treated with Trim33 siRNA vs control siRNA before (day 0, myoblast) and after (day 3, myotubes) differentiation. q-values compare the expression levels of KO vs. WT samples. Each dot corresponds to an individual sample measurement. * < 0.05, ** < 0.01, *** < 0.001.

To determine whether Trim33 KO affects the skeletal muscle transcriptome, we analyzed RNAseq data obtained from mouse muscles harvested before and after muscle injury. Surprisingly, cholecystokinin (CCK) gene expression was the only gene with significant differential expression in KO compared to WT mouse muscle. Specifically, while CCK expression levels were similar in Trim33 KO and WT mice before cardiotoxin injection (log2FC = 0.96, q = 0.3), they were markedly increased in Trim33 KO muscle at day 3 (log2FC = 5.67, q = 0.0002), day 5 (log2FC = 6.63, q = 7.1•10^–8^), day 10 (log2FC = 6.14, q = 3.8•10^−5^), and day 14 (log2FC = 5.52, q = 0.0003) after muscle injury. CCK levels returned to normal baseline levels at day 28 following muscle injury (log2FC = 1.4, q = 1) (Fig. 3). These results were confirmed by qPCR (Supplementary Fig. 4).

To explore CCK expression in muscle further, we analyzed KO versus WT satellite cells. Undifferentiated KO and WT satellite cells (myoblasts) had similar CCK expression levels. However, after two days in differentiation media (myotubes), CCK levels increased in KO compared to WT satellite cells (log2FC = 2.3, p = 0.05) (Fig. 3). We found that CCK levels were also upregulated in differentiated C2C12 mouse muscle cells (myotubes) treated with Trim33 siRNA compared to control siRNA (log2FC = 10.4, q = 1.9•10^−57^) (Fig. 3).

## Discussion

The role of Trim33 in development, growth, and regeneration is known to be tissue-specific, and its role in muscle growth and regeneration has been largely unexplored. Trim33’s classic role as a negative regulator of the TGFβ pathway and the involvement of the TGFβ pathway in muscle regeneration led us to hypothesize that Trim33 might be involved in muscle development and/or regeneration. In the current study, we created conditional knock out Trim33 mice to test this hypothesis.

Although Trim33 KO mice were modestly smaller than WT mice, neither muscle development nor muscle regeneration appeared to be impaired in the absence of Trim33. Unexpectedly, we did find a striking overexpression of CCK in Trim33 KO muscle, as well as in satellite cells and C2C12 cells in which Trim33 expression was reduced.

CCK is a neuropeptide expressed in the small intestine as well as in the central and peripheral nervous system with multiple relevant physiological roles including regulating digestion^11,12^, insulin secretion^13,14^, satiety^15,16^ and anxiety^17^. While the effect of CCK on its receptors is well-studied, the factors that regulate CCK expression have not been well-defined^18^. Our results here suggest that Trim33 plays a role in inhibiting CCK expression. Furthermore, we speculate that manipulating this pathway could have important therapeutic implications in the treatment of diseases such as diabetes, obesity or anxiety given the biologic effects of CCK in the intestine and central nervous system.

The link between TRIM33 and CCK remains elusive. Although it has been shown that CCK expression may be suppressed by TGFβ^19^, we could not find any effect of decreased TRIM33 expression on the TGFβ pathway in skeletal muscle to directly link TRIM33 to CCK.

This study has a series of limitations. First, as our primary objective was to study the effect of Trim33 KO during muscle differentiation, we focused our experiments in muscle cells and tissue and did not analyze the effect of Trim33 KO in other relevant tissues with known CCK expressions, such as the small intestine or the brain. Second, even though we demonstrated that the expression of CCK is increased by knocking-out/down Trim33, we did not analyze the posttranscriptional modifications of the CCK protein, and, thus, it is possible that the resulting muscle protein is not functional. Finally, in these studies, we achieved reduction ranging from 40–60% in Trim33 expression both in muscle, satellite cells, and C2C12 cells (Supplementary Fig. 5). Although this reduction was sufficient to dis-inhibit CCK expression, it may have been insufficient to produce abnormalities in muscle development and regeneration or in the TGFβ pathway.

In summary, while we could not find an effect of Trim33 reduction in muscle differentiation or in the TGFβ pathway, we incidentally found strong evidence that Trim33 may play a role in suppressing the expression of CCK, a key neuropeptide with multiple physiological functions both in the gut and in the central nervous system.

## Supplementary information

Supplementary information
