# Supplementary material for: Trim33 (Tif1γ) is not required for skeletal muscle development or regeneration but suppresses cholecystokinin expression
Source: Sci Rep. 2019 Dec 6;9:18507. doi: 10.1038/s41598-019-54651-8 (PMC6898130; doi:10.1038/s41598-019-54651-8)
Supplement: Supplementary file 1 — Supplementary information [file 41598_2019_54651_MOESM1_ESM.docx]

**SUPPLEMENTARY INFORMATION**

**Trim33 (Tif1γ)** **is not required for skeletal muscle development or regeneration but suppresses cholecystokinin expression.**

Cassie Parks^1^*, Katherine Pak^1^*, Iago Pinal-Fernandez^1,2,3^*, Wilson Huang^1^, Assia Derfoul^1^, Andrew L Mammen^1,2^

**Supplementary Figure 1. a)** Amplification of null allele in Trim33 KO mouse (Pax7-Cre; Trim33^F/F^), and control WT mouse (Trim33^F/F^ littermate). Expected size = 362 bp. **b)** Sanger sequencing of null allele using forward (top) and reverse (bottom) primers.

**Supplementary Table 1.** Complete information of the antibodies used for satellite cell FACS.

| **Antibody** | **Catalog #** | **Source** | **Host** | **Application** | **Dilution** |
| --- | --- | --- | --- | --- | --- |
| a7-integrin-PE | K0046-5 | MBL | Mouse | FACS | 1:100-1:200 |
| Sca-1-FITC | 557405 | BD | Mouse | FACS | 1;200 |
| CD 11b-FITC | 553310 | BD | Rat | FACS | 1;200 |
| CD 31-FITC | 553372 | BD | Rat monoclonal | FACS | 1;200 |
| CD 45-FITC | 553080 | BD | Rat | FACS | 1;200 |

**Supplementary Figure 2. Normal pattern of muscle gene expression during regeneration in Trim33 KO mice.** Evolution of the expression levels (log_2_[TPM+1]) after cardiotoxin injection of key markers of muscle differentiation (NCAM1, MYOG, MYOD1, PAX7, MYH3, MYH8) and muscle structural proteins (ACTA1, MYH1, MYH2). We did not detect significant differences in the expression of these genes between Trim33 WT and KO mice.

**Supplementary Figure 3. Normal pattern of TGFβ pathway gene expression during regeneration in Trim33 KO mice.** Evolution of the expression levels (log_2_[TPM+1]) after cardiotoxin injection of key effectors of the TGFβ pathway (SMAD4, SP1, MYC, CDKN2B, EP300, RBL1, E2F2, E2F5, PITX2). We did not detect significant differences in the expression of these genes between Trim33 WT and KO mice.

**Supplementary Figure 4**. qPCR fold-change expression levels of cholecystokinin (CCK) in Trim33 KO vs. WT mice muscle after cardiotoxin injection showing a marked increase of the CCK expression in KO mice during muscle regeneration.

**Supplementary Figure 5**. RNAseq fold-change reduction in the expression levels of TRIM33 before (day 0) and 3 days after cardiotoxin injury in the skeletal muscle of WT vs KO mice, in satellite cells harvested from WT vs. Trim33 KO mice before (day 0) and after (day 3) differentiation, and in C2C12 cells treated with Trim33 siRNA vs control siRNA before (day 0) and after (day 3) differentiation into myotubes.
